# Medically Unexplained Physical Symptoms in Hospitalized Patients: A 9-Year Retrospective Observational Study

**DOI:** 10.3389/fpsyt.2018.00626

**Published:** 2018-11-23

**Authors:** Nicola Poloni, Marta Ielmini, Ivano Caselli, Francesca Ceccon, Lucia Bianchi, Celeste Isella, Camilla Callegari

**Affiliations:** Section of Psychiatry, Department of Medicine and Surgery, University of Insubria, Varese, Italy

**Keywords:** somatic symptoms disorders, Medically Unexplained Physical Symptoms, psychosomatic medicine, consultative psychiatry, psychosocial interventions

## Abstract

**Introduction:** “Medically Unexplained Physical Symptoms” (MUPS) defines a subgroup of patients presenting physical symptoms of unclear origin. The study aims to profile clinical and socio-demographic characteristics of patients with MUPS.

**Materials and Methods:** This 9-years observational retrospective study assesses all patients admitted between 2008 and 2016 in the divisions of neurology and gastroenterology. Socio-demographic and clinical variables were evaluated: gender, age, diagnosis or diagnostic hypothesis, presence of psychiatric comorbidities, psychiatric evaluation, pharmacological treatment, number of admissions/visits.

**Results:** Among 2,479 neurological patients 10.1% presented MUPS. Patients were more frequently women (63.5%), with a mean age of about 50 years. Reported symptoms were headache (22.6%), seizures (8.7%), vertigo (5.9%), fibromyalgia (5.5%), paresthesia (5.1%), visual disturbances (5.1%), amnesia (3.9%). The diagnosis was somatoform disorder in 6.3% of cases, conversion disorder in 2.7%, and somatic symptom disorder in 1.5% only. 2,560 outpatients were evaluated in gastroenterology division. 9.6% (*n* = 248) of patients had MUPS; 62.1% of them were women. The most affected age group ranged between 15 and 45 years. The most frequent diagnoses were functional abdominal pain (50%), dysmotility-like dyspepsia (26.6%), irritable bowel syndrome (10.4%), meteorism of unknown cause (2.4%), hiccup (1.6%), burning mouth syndrome (1.2%). No patients received a diagnosis of somatic symptom disorder.

**Discussion:** Patients with MUPS are more often women, of middle age, with self-referred specific symptomatology. While neurological patients received a diagnostic-therapeutic approach in line with the literature, gastroenterological patients mainly received antipsychotics. A more comprehensive assessment and a development of psychoeducational interventions are needed to improve patients' quality and quantity of life.

## Introduction

The term MUPS (Medically Unexplained Physical Symptoms) is used as umbrella term to refer to conditions defined by physical symptoms not better defined by another disorder (1, 2). MUPS are extremely common, accounting for 15–30% of primary care patients and around half of secondary care patients (3–5).

MUPS can be partially explained by a complex interaction of physiological and psychological factors (1, 6) and they can cause disability persisting for at least 1 year in up to 30% of patients (3), affecting daily functioning, interfering with work productivity, and resulting in use of healthcare resources than in other patient groups (2, 7, 8).

Relations between physicians and patients with MUPS are often strained. Physicians often perceive patients with MUPS as difficult, frustrating, and demanding (9). Therefore, MUPS can be a challenging experience for clinicians with a high risk to develop symptoms of burn out (10). At the same time, patients with MUPS report feeling dissatisfied, disbelieved and dismissed by clinicians (1).

In the *Diagnostic and Statistical Manual of Mental Disorders, Fourth Edition, text revised* (*DSM-IV-TR*) the presence of MUPS was a criterion for the diagnosis of a somatic symptom disorder. In DSM-5 the somatoform disorders were replaced by the somatic symptom disorder, consisting of a new set of criteria including positive psychological ones (11, 12). The most important change was the focus on the importance of physical symptoms associated with significant distress and impairment (1). However, van Dessel et al. found a strong association between the presence of positive psychological criteria in DSM-5 and symptoms severity and physical functioning in patients with MUPS (13). Moreover, Huang et al. discussed the importance of recruiting settings, the comorbidity of pain disorder and undifferentiated somatoform disorder, and the impact of cultural factors in somatic symptoms disorder's diagnosis (14).

Some factors have been recognized as predictors of complexity of the clinical picture: high number of somatic symptoms, psychiatric comorbidity (particularly, depressive or anxious disorders), psycho-social risk factors such as history of child abuse or violence (8, 15).

The most frequently symptoms reported by patients are pain, fatigue, neurological, gastrointestinal, dermatological, and rheumatological symptoms. Although in most cases these symptoms resolve spontaneously (16), a small but significant percentage may worsen over time (8, 17–19).

Early detection of risk factors for the most complex or persistent types of MUPS is essential to provide adequate support to these patients (15). In view of the difficulties in the management of these disorders and of their impact on patients' quality of life, this 9-year retrospective observational study aims to profile clinical and socio-demographic characteristics of patients with MUPS and to identify the most frequent diagnoses of somatic complaints' disorders formulated in neurology and gastroenterology, and their clinical approach.

## Materials and methods

In this observational and retrospective study all patients admitted between 2008 and 2016 in the divisions of neurology and gastroenterology in a teaching hospital in Northern Italy were assessed. Gastroenterology patients' data were available from 2011, year when the outpatient clinic was established.

The teaching hospital includes several hospital wards and outpatient clinics. MI, IC, LB, and FC were four Psychiatry Section clinicians (also authors of the manuscript) enrolled as investigator to collect data from hospital databases not directly involved in analyzed patients' treatment.

Data from patients presenting the following inclusion criteria were used: age ≥18 years, be an inpatient (neurology) or an outpatient (gastroenterology) of the teaching hospital; present symptoms with apparently no medical cause, or whose cause remains unclear; have a diagnosis of *Somatoform Disorder* or *Somatic Symptoms and Related Disorders* (according to *DSM-IV-TR* or *DSM-5* criteria, through ICD code Conversion Table; Italian statistical medical recoding system is *ICD*); present all instrumental and laboratory examinations clean.

Data from patients presenting only a psychiatric history were excluded. Patients' data were made anonymous obscuring sensitive data in the research to protect the recognizability of the patients. Moreover, data were anonymous because registered in electronic dataset and used in a collective form. The following socio-demographic and clinical variables were evaluated: gender, age, diagnosis or diagnostic hypothesis, presence of previous or concurrent psychiatric comorbidities, psychiatric evaluation, pharmacological treatment, number of admissions/visits. Patients data were collected through the medical records and the reports of the visits.

As data were made anonymous and unidentifiable, the Provincial Health Ethical Review Board (Ethics Committee of Insubria—*Azienda Socio Sanitaria Territoriale Sette Laghi*, Varese, Italy) consulted prior to the beginning of the study, has confirmed that, as it was a retrospective study, it did not need authorization from the Board.

Descriptive analyses, which include means, standard deviation and demographic variables percentages, were used to summarize epidemiological and clinical characteristics. Fisher exact test at two-tailed was used to evaluate MUPS distribution between the two groups of patients and between genders. Significance threshold was set at *p*-value < 0.05. Data analyses were performed using the IBM^®^ SPSS^®^ Statistics, version 22.0.

## Results

Sociodemographic and clinical data are reported in Table 1.

**Table 1:** Socio-demographic and clinical characteristics.

	**Neurology Inpatients *N* (%)**	**Gastroenterology Outpatients *N* (%)**
Total number	2,479 (100%)	2,560 (100%)
Presence of MUPS	252 (10.1%)	247 (9.6%)
**GENDER**		
Men	92 (36.5%)	93 (37.5%)
Women	160 (63.5%)	154 (62.1%)
Mean age (years)	47	40
Men	50	35
Women	46	42

### Neurology patients

Data from 2,479 patients admitted to Neurology ward were evaluated and 252 (10%) presented MUPS; among them 63.5% were women. No statistically significant difference of MUPS distribution between genders emerged (*p* = 0.88). The most affected age group ranged from 36 to 45 years, while the middle age was 46 years for women and 50 years for men.

47.2% of patients had a clinical history of psychiatric disorder or a concomitant mood disorder. Reported symptoms were headache (22.6%), seizures (8.7%), vertigo (5.9%), fibromyalgia (5.5%), paresthesia (5.1%), visual disturbances (5.1%), amnesia (3.9%). The diagnosis was somatoform disorder in 6.3% of cases (*n* = 16), conversion disorder in 2.7% (*n* = 7), and somatic symptom disorder in 1.5% (*n* = 4). Furthermore, in 41.6% of cases, to emphasize the functional origin of the symptoms, it was declared that neuro-radiological and neurophysiological findings were all negative.

**Figure 1:**
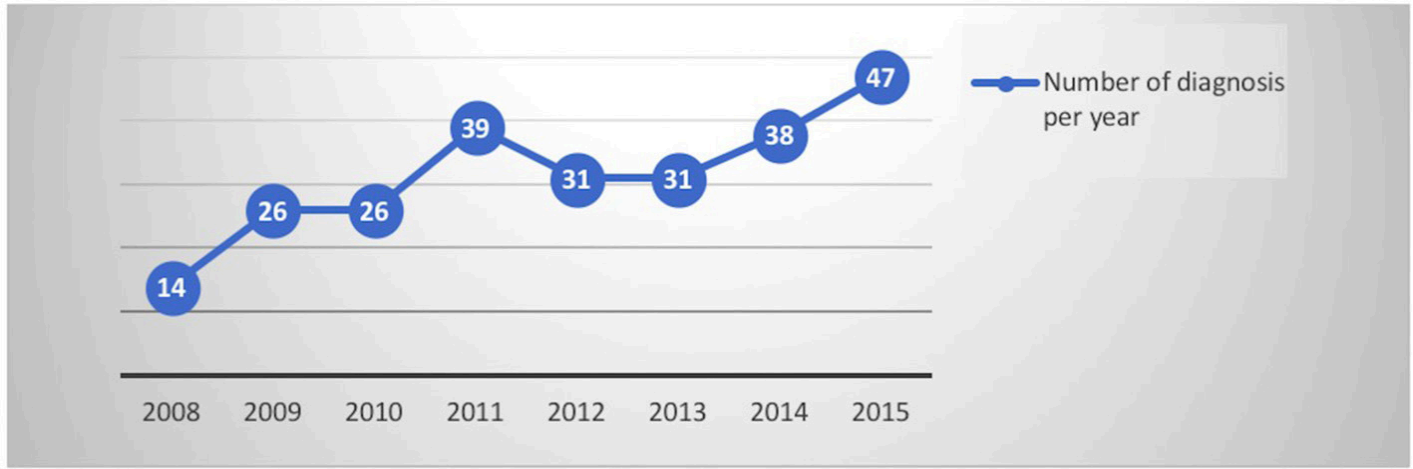
Number of diagnosis per year.

**Figure 2:**
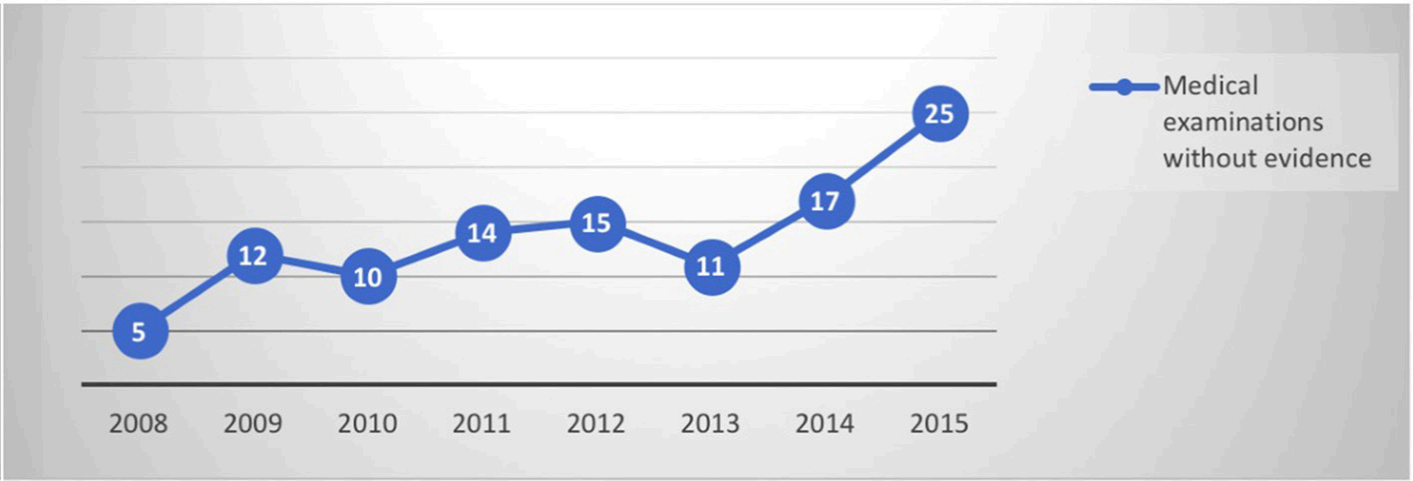
Medical examinations without evidence per year.

By evaluating the trend of data collected per year, it is interesting to note that the diagnoses have increased concomitantly with an increasing number of neuro-imaging negative assessments (as shown in Figures 1, 2). The number of psychiatric consultations requested during hospitalization and the number of patients sent to the psychiatrist in the post hospitalization are unchanged over the years.

Although women are more represented than men, the medical examinations without evidences are comparable between genders, as well as psychiatric anamnesis and number of patients sent to psychologist or psychiatrist.

On the other hand, the presentation of symptoms appears to be different between the two genders: men have manifested the disorder in the form of paresthesia (17.3%) and epileptic crisis (15.2%); women lamented predominantly headache (30%), paresthesia (16.2%), epileptic crisis (10%), and vertigo (8%).

During hospitalization, a psychiatric consultation was requested for 24.6% of patients and for 21.8% of patients a continuation of care by psychiatric services was suggested; 42.8% of them received a treatment with a SSRI (35.1%) or a SNRI (7.7%); antipsychotics were prescribed in 9.9% of cases. Thirteen percent of patients received only benzodiazepine.

### Gastroenterology patients

Two thousand five hundred and sixty outpatients were evaluated in gastroenterology division. 9.6% (*n* = 248) of patients had MUPS, without significative statistical difference compared to MUPS distribution among neurological patients (chi-square 0.32; *p* = 0.57); 62.1% of them were women. No statistically significant difference of MUPS distribution between genders emerged (*p* = 0.19).

The most affected age group ranged between 15 and 45 years. Average age was 35 years for men and 42 years for women.

Regarding gastroenterology patients: 22.9% of patients had a positive psychiatric history or a concomitant mood disorder. The most frequent diagnoses were functional abdominal pain (50%), dysmotility-like dyspepsia (26.6%), irritable bowel syndrome (10.4%), meteorism of unknown cause (2.4%), hiccup (1.6%), burning mouth syndrome (1.2%). No patient had received a diagnosis of somatic symptom disorder. During the assessment, a psychiatric visit was recommended in 1.6% of cases only.

Evaluating the diagnoses made per year, differently from neurological patients, an increase in data did not emerge, as for the negative investigations (as shown in Figures 1, 2). The differences between the two genders seemed to be related to the anamnestic background: a greater presentation of previous or concurrent psychiatric disorders emerged among women.

Symptoms complained by patients in both genders are overlapping: dysmotility (23% among men and 28% among women), abdominal pain (41.9% among men and 27% among women), and irritable bowel syndrome (11.8% among men and 11.6% among women). The major differences related to symptoms between genders were dyspepsia, persistent hiccups, and jugular constriction more represented among men; vice versa burning mouth syndrome and hot flashes with chest-pain have been observed only among women.

Symptoms in gastroenterology group were not usually assessed through exams but were treated with Levosulpiride (38.7%) and antispasmodic (33.8%); only 8.1% of patients received an antidepressant (SSRI in 5.6% and or SNRI in 2.6% of patients) and only 1.6% of patients was sent to psychiatric care.

## Discussion

The notion that most MUPS are the result of a single process of somatization is no longer supported by the evidence. Physiology, personality traits, life experiences, health cognition, and interaction with healthcare professionals, in fact are all important in the development of medically unexplained symptoms, and a new model useful to understand MUPS is that of a complex adaptive system (20, 21).

Starting from patients' characteristics it is interesting to note that a significant number of patients visiting these hospital departments presents medical unexplained symptoms. This datum is partially in line with the literature showing higher percentage of patients with MUPS among general practice (25–50% of patients) and similar percentage in emergency department (18.5% of non-trauma patients visiting emergency departments) (22, 23).

According to the literature, patients with MUPS are more often women, of middle age, with self-referred specific symptomatology (23). In the neurology ward patients present a psychiatric history more frequently than in gastroenterology. From the largest study on comorbidity rates emerged that 50.6% of patients suffering from MUPS has a personality disorder. Other authors showed that MUPS are often associated to affective disorders, primarily depression, and especially in later life (24, 25). The role of inflammation in major depressive disorder (MDD) and the impact of exposure to early stressful events in increasing the vulnerability to develop psychopathologies may represent a possible common ground in developing physical symptoms, involving similar underlying pathogenic mechanisms (26–28). Additionally, the co-existence of MUPS and medical explained conditions in the same individuals tends to result in the exclusion of the MUPS episodes from the appropriate diagnostic code (29, 30).

Dealing with the diagnostic approach of somatic symptoms disorders, instead of neurological inpatients, among gastroenterology's outpatients no one received a diagnosis of somatic symptom disorder; this difference emphasizes the greater attention to psychopathological problems given in a department such as neurology.

Despite the numerous medical exams required, a psychiatric consult was prescribed in a minority of patients, remaining unchanged over the years in the whole sample. While neurological patients received a diagnostic-therapeutic approach in line with the literature, gastroenterological patients mainly received antipsychotics. In literature emerged that SSRIs are preferred, in monotherapy or in association with atypical antipsychotics (12, 31). However, from a Cochrane review emerged that the efficacy of new generation antidepressants has to be balanced with the long-term side effects that amplify the perception of somatic symptoms (32–34).

Psychotherapeutic treatments are varied; in literature emerged that approaches such as CBT can be helpful for these patients (35). However, practitioners often have not inadequate service opportunities (3). Focusing on the treatment of a psychiatric disorder can be useful for the resolution of physical symptoms (36). Different strategies, based on patient' risk profiles, as detected by consensus and expert opinion in the guideline working group, could be used in order to treat patients and reduce MUPS associated healthcare costs. In the Dutch Multidisciplinary Guideline for MUPS and Somatoform Disorder (SD), a disease management approach is recommended in which risk profiles are defined and a stepped care algorithm for treatment is described; in high risk patients a multidisciplinary team treatment is recommended (9, 36, 37). However, more researches are needed for validation of screening instruments for MUPS and SD, for preventive psychosocial interventions aimed at improvement of the patient-doctor relationship and at reduction of healthcare costs.

### Strength and limitations

The strength is the evaluation of a high number of data starting from a medical approach. Despite this point, retrospective data do not provide information on follow-up. Moreover, diagnosis of somatoform disorder or somatic symptom disorder was formulated by non-specialists. There are no data about costs; this point could represent a future goal for a study about psychoeducation and psychosocial interventions.

## Author contributions

NP and CC conceptualized and wrote the first draft of the manuscript. MI, IC, LB, and FC contributed with commentaries and suggestions and collected the data. CI wrote the references. MI, IC, and CC also reviewed and supervised all the writing process.

### Conflict of interest statement

The authors declare that the research was conducted in the absence of any commercial or financial relationships that could be construed as a potential conflict of interest.
